# Effect of a four-week ketogenic diet on exercise metabolism in CrossFit-trained athletes

**DOI:** 10.1186/s12970-019-0284-9

**Published:** 2019-04-05

**Authors:** Krzysztof Durkalec-Michalski, Paulina M. Nowaczyk, Katarzyna Siedzik

**Affiliations:** 1 Institute of Human Nutrition and Dietetics, Poznan University of Life Sciences, Wojska Polskiego 31, 60-624 Poznań, Poland; 2 Department of Food and Nutrition, Poznan University of Physical Education, 61-871 Poznań, Poland

**Keywords:** Ketogenic diet, CrossFit, Nutritional intervention, Energy substrates, Exercise metabolism, Sport

## Abstract

**Background:**

The ketogenic diet is becoming a popular nutritional model among athletes. However, the relationship between its use and metabolism during exercise seems to have not been fully investigated.

**Methods:**

The aim of the study was to assess the effects of a four-week ketogenic diet (KD) on fat and carbohydrate (CHO) utilization during an incremental cycling test (ICT) in CrossFit-trained female (*n* = 11) and male (n = 11) athletes. During the ICT (while consuming the customary diet and after the KD), oxygen uptake and carbon dioxide exhalation were registered, and CHO and fat utilization as well as energy expenditure were calculated.

**Results:**

In males, the KD led to an increase in fat utilization (g·min^− 1^·kg_FFM_^− 1^ and % oxidation). It was particularly noticeable at exercise intensities up to 80% of VO_2max_. An increase in the area under the curve (AUC) was seen in males but not in females at up to ≤65% VO_2max_ of fat utilization.

**Conclusions:**

Male CrossFit-trained athletes seem to be more prone to shifts in macronutrient utilization (in favor of fat utilization) during submaximal intensity exercise under a ketogenic diet than are female athletes.

**Trial registration:**

Clinical Trials Gov, NCT03665948. Registered 11 September 2018 (retrospectively registered).

**Electronic supplementary material:**

The online version of this article (10.1186/s12970-019-0284-9) contains supplementary material, which is available to authorized users.

## Background

Carbohydrates (CHO) and fat are the main sources of fuel oxidized in muscles during exercise [1]. In contrast to fat, endogenous stores of CHO are limited. The storage form of carbohydrate, glycogen, is found almost exclusively in muscle and liver and represents only ~ 8000 kJ (about 1911 kcal) in untrained individuals and about 20–50% higher in trained men and women. However, there is a vast quantity of fat stored even in the leanest of athletes (approximately 600,000 kJ) [2]. Physical performance (mainly endurance) and exercise ability can be limited when endogenous CHO are the dominant fuel [3]. A high CHO diet has been traditionally promoted for athletes in order to maximize muscle and liver glycogen stores, as well as the ability to maintain their effective utilization, which often determines the final effectiveness of physical exercise [4]. However, over the last two decades, reports suggesting benefits from purposely and strategically reducing the availability of CHO during some or all of an athlete’s training sessions (e.g., low CHO high fat diet, high/low CHO periodization, fed/fasted training) has appeared with increased frequency in the literature [5–8]. These dietary regimes are supposed to optimize fuel mobilization and utilization during exercise by activating fatty acids as an energy source during exercise.

The ketogenic diet (KD) is a special example of a low CHO high fat diet. The original KD was designed with a 4:1 lipid:non-lipid ratio with ~ 75–80% of the daily energy intake from fat, 15% from protein (PRO), and less than 5% and/or 20–50 g CHO per day [3, 9–11]. After several days of drastically reduced CHO intake while maintaining the usual energy intake, glucose reserves become depleted and are no longer sufficient for either normal fat oxidation (via oxaloacetate in the Krebs cycle) or to supply energy to the brain and central nervous system [10]. It is worth mentioning that the first studies on KD focused mainly on its efficacy in treating epilepsy and came from the early 1930s [12]. Its possible efficacy was further investigated with regard to weight loss [13], insulin resistance, diabetes or high blood pressure [14].

The KD can be characterized by pleiotropic activity. The significant influence of the KD may be related to its impact on the adaptation of the body through the regulation of the molecular mechanisms of cellular signaling [11, 15–17]. Activation of these signaling pathways may lead to a significant increase in the physical and exercise capacity, by stimulating, e.g., mitochondrial biogenesis, capillarization, regeneration processes, and especially, effective fat energy substrate utilization [18–20]. The relationship between KD and inflammation/oxidative stress may also be significant, which has been observed in the therapy of metabolic, neurodegenerative, and other disorders related to the severity of inflammation (e.g., diabetes and obesity, polycystic ovary syndrome, autism, acne, asthma, multiple sclerosis, Alzheimer’s and Parkinson’s diseases) [11, 15, 16, 21–30]. Beneficial effects may also involve the strengthening of brain function and cognitive performance [18, 20, 31].

However, the main mechanism of influence of the KD on the human body is its impact on metabolic reorganization [11, 16, 32–35]. Due to the lack of optimal CHO availability, free fatty acid (from the diet or adipose tissue) oxidation and ketogenic amino acid degradation result in the formation of ketone bodies (KB; β-hydroxybutyrate, acetoacetate, and acetone) in the mitochondrial matrix of liver cells [11, 16]. After transporting the KB to extrahepatic cells, they can be used as an energy substrate (acetyl-CoA) in the production of ATP through the oxidative phosphorylation pathway in the citric acid cycle (TCA) [16].

In addition, the metabolic effects of KB are also associated with the sparing and/or attenuation of fuel (glycogen, glucose) selection and lactate production during exercise, anti-lipolytic effects, and increased reliance on intramuscular triglycerides [11, 17]. Further studies are needed to demonstrate the impact of the KD on the synthesis/proteolysis of muscle proteins, although it now seems that anabolic pathways may be downregulated in individuals following a KD diet [36–38]. It can therefore be concluded that diets high in fat upregulate the release, transport, uptake, and utilization of fat in the muscle, even in endurance athletes whose training would have been expected to maximize such adaptations [39–41].

The effectiveness of KD in supporting weight reduction has made it quite popular among athletes competing in sport disciplines with weight categories or aesthetic aspects [42, 43]. New lines of research concerning the utility of KD in sports have focused on performance in endurance trained athletes, most of which concurrently addressed the issue of energy metabolism during exercise and revealed increased fat oxidation under KD [3, 41, 44–46]. However, few studies have investigated the relations between exercise intensity (as measured by maximal oxygen uptake) and fat utilization in detail and these have been underpowered due to low sample sizes [46]. Moreover, no between-gender comparisons in possible energy metabolism differences related to the KD have been made. However, it is known that there are gender differences in whole-body fat oxidation kinetics during exercise. Fat oxidation rates and maximal fat oxidation at submaximal intensities are greater in women than in men, and maximal fat oxidation occur at substantially higher exercise intensities in women than in men [47].

Based on the above facts, we hypothesized that in actively training male and female CrossFit practitioners, a KD for 4 weeks would induce shifts in fuel utilization during exercise in favor of fat utilization. Our secondary hypotheses were that changes in fat utilization under the KD would differ between exercise intensities (as measured by %VO_2max_) and between gender. To test these hypotheses, we conducted a single arm nutritional intervention trial in a group of CrossFit athletes (11 females and 11 males) consuming a KD (≥75% of daily energy from fat, 1.7 g of PRO per kilogram of body mass, and up to 5% energy from CHO) for 4 weeks.

## Methods

### Participants

**Fig. 1:**
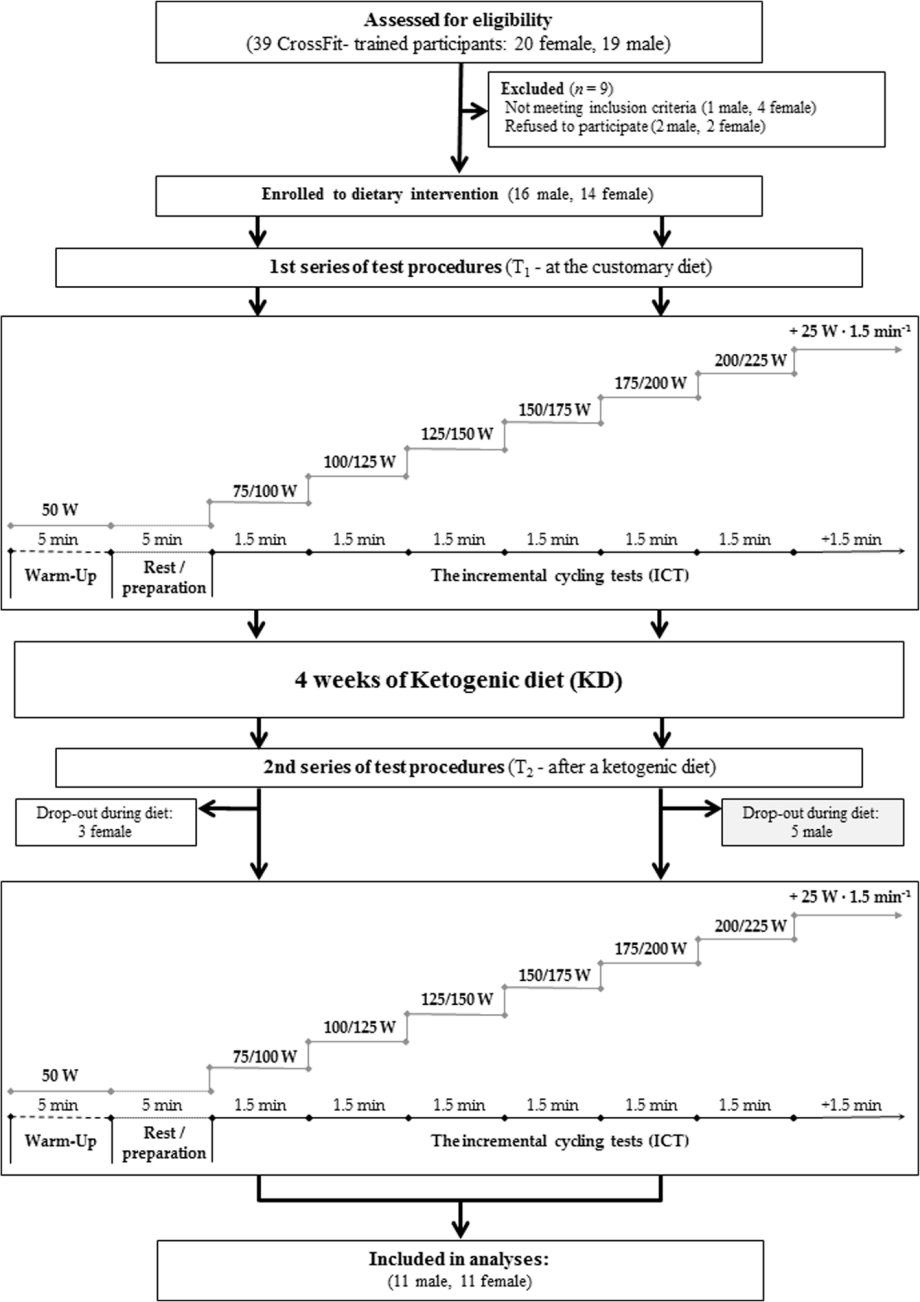
A flow chart of the study design

Thirty participants were initially enrolled in this study. However, 22 (11 women, 11 men; mean ± SD: age 29.5 ± 4.4 years, body height 172 ± 8 cm, baseline values of body mass 71.1 ± 12.8 kg, and maximal oxygen uptake 45.8 ± 7.5 mL·min^− 1^ kg^− 1^) completed the entire study protocol and were included in the analyses (Fig. 1, Table 1). The participants were recreationally and regularly training for CrossFit at the Caffeine Barbell, Hangar, Reebok CrossFit Poznań and Rankor Athletics clubs in Poznań, Poland. The criteria for qualifying for the study, included being aged from 18 to 40 years, in good health with a valid and up-to-date medical certificate confirming the athlete’s ability to practice sports, at least 2 years of regular CrossFit training experience, and a minimum of 4 workout sessions (CrossFit) per week. We included both males and females because of the equal participation of both genders in CrossFit training, as well as because of the willingness to assess whether the KD has an impact related to the gender of the subjects undergoing this type of training.

**Table 1:** Anthropometric characteristics

		All	Females	Males
*n*	(years)	22	11	11
Age		29.5 ± 4.4	30.5 ± 3.3	28.5 ± 5.3
Body height	(cm)	172 ± 8	166 ± 7	177 ± 5
Body mass	(kg)			
	Customary diet	71.1 ± 12.8	60.8 ± 4.9	81.3 ± 9.5
	Ketogenic diet	70.1 ± 12.6	60.0 ± 4.4	80.1 ± 9.7
		NS	NS	NS
VO_2max_	(mL·min^− 1^· kg^− 1^)			
	Customary diet	45.8 ± 7.5	43.1 ± 7.1	48.6 ± 7.7
	Ketogenic diet	44.9 ± 7.6	41.1 ± 5.9	48.8 ± 7.4
		NS	NS	NS
	(mL·min^−1^)			
	Customary diet	3269 ± 848	2597 ± 391	3941 ± 604
	Ketogenic diet	3168 ± 846	2463 ± 356	3874 ± 530
		NS	NS	NS

*Note: Values are expressed as means ± standard deviation (SD)*. *NS: not significant*

Exclusion criteria included being a current smoker or participating in illicit drug use, alcohol consumption greater than 1–2 drinks/week, and dietary supplement use or being on any special diet within 3 weeks of the study’s commencement. For females, additional exclusion criteria were being pregnant or planning to become pregnant during the study or the presence of a menstrual cycle disorder.

The primary recruitment strategy was contacting certified Crossfit trainers responsible for managing training sessions in Crossfit clubs located in the Wielkopolska region. They assisted with the identification, inclusion, and confirmation of the aspects declared by the participants related to the inclusion criteria (such as training experience and number of sessions per week). They also supported the control of training and diet in their “Crossfit boxes” during the tests and also participated in our research.

The study protocol was reviewed and approved by the local institutional review board (Bioethics Committee at Poznań University of Medical Sciences, reference numbers: 681/16 and 683/16, 10 November 2016). Written informed consent was obtained from all participants before the study began. All procedures were conducted in accordance with the ethical standards of the 1964 Helsinki Declaration. The trial was conducted from January to December 2017. This trial was registered at Clinical Trials Gov (website: https://www.clinicaltrials.gov/ct2/show/NCT03665948; Clinical Trial Identification Number: NCT03665948). The study was registered retrospectively since registration was not required when the study enrolment started. The authors confirm that all ongoing and related trials for this intervention are registered.

### Study design and protocol

The study protocol included three visits to the laboratory (Fig. 1). The first visit (T_0_) consisted of familiarization to the progressive cycling test, food recording method, physical activity questionnaire and the study protocol for the physical capacity test as well as anthropometric measurements. After enrollment, subjects began a 14-day run-in phase during which they ate their customary diet (CD) and recorded all ingested food and beverages in a food diary (Table 2, Additional file 1: Table S1). Food intake data were analyzed and used to determine each subject’s general and customary eating habits. During this period, total daily energy expenditure (EE) was estimated. After the run-in phase, the first laboratory study visit was conducted (T_1_) during which an incremental cycling test was performed (Fig. 1).

**Table 2:** Nutritional value of customary and ketogenic diets

		Females	Males
Energy (kcal·day^− 1^)	Customary diet	2485 ± 279	2645 ± 289
	Ketogenic diet	2659 ± 273	2955 ± 265
Protein (g·day^− 1^)	Customary diet	109 ± 26	127 ± 15
	Ketogenic diet	115 ± 17	134 ± 9
Protein (g·kg_BM_·day^− 1^)	Customary diet	1.8 ± 0.4	1.6 ± 0.2
	Ketogenic diet	1.9 ± 0.2	1.7 ± 0.2
Protein (% _energy intake_)	Customary diet	18 ± 5	19 ± 3
	Ketogenic diet	17 ± 2	18 ± 3
Fat (g·day^−1^)	Customary diet	101 ± 19	107 ± 29
	Ketogenic diet	230 ± 24*	245 ± 29*
Fat (g·kg_BM_·day^−1^)	Customary diet	1.7 ± 0.4	1.3 ± 0.3
	Ketogenic diet	3.8 ± 0.5*	3.2 ± 0.6*
Fat (% _energy intake_)	Customary diet	37 ± 7	36 ± 8
	Ketogenic diet	78 ± 2*	77 ± 2*
Carbohydrate (g·day^−1^)	Customary diet	285 ± 79	294 ± 80
	Ketogenic diet	33 ± 3*	33 ± 5*
Carbohydrate (g·kg_BM_·day^−1^)	Customary diet	4.7 ± 1.5	3.6 ± 0.9
	Ketogenic diet	0.5 ± 0.1*	0.4 ± 0.1*
Carbohydrate (% _energy intake_)	Customary diet	45 ± 10	44 ± 9
	Ketogenic diet	5 ± 0*	4 ± 1*

*Note: Values are expressed as means ± SD*. * significantly different from customary diet (p < 0.05)

On the next day, after the baseline T_1_ testing, the subjects started the dietary intervention (Fig. 1). Throughout the KD intervention period, participants kept a nutritional diary to assess each individual’s diet compliance with the study diet. After the participants had completed a four-week experimental KD intervention, the second series of laboratory study visits (T_2_) was conducted. Exercise tests were performed twice, before (at the CD) and after the KD diet (T_1–2_), in the laboratory. During the exercise tests, substrate oxidation rates and EE were evaluated. The subjects were asked to maintain a similar training load and the same physical activity level throughout the study.

### Study visits

The protocol included two study visits to the laboratory: before (T_1_, at the CD) and after (T_2_) the KD diet (Fig. 1). At each visit, body mass and composition were measured, followed by performance of the exercise tests. Three hours before the T_1_ and T_2_ visits, the participants consumed a standardized meal corresponding to their habitual food intake or to menu of the KD diet, respectively. The three-hour period between the meal and exercise was chosen to prevent potential short-term effects of a meal on exercise performance [48–50]. On the day following T_1_ visit, the participants began their prescribed four-week KD diet ending with the T_2_ visit.

### Study diets

The KD model was individualized to ensure weight maintenance throughout the dietary interventions and diets were energetically normalized (covered the estimated EE). They assumed coverage of the recommended daily energy requirement of up to 5% of energy from CHO [3, 9–11]. PRO were administered in the amount of 1.7 g per kilogram of body mass. The remaining energy needs were provided by fats (fats provided more than 75% of the daily energy requirement). An appropriate supply of essential fatty acids in the diet was ensured. Each of the subjects in study group received 10-day menus. Diets were developed considering the type of foods, cooking methods, and pre-established macronutrient ratios. For all diets, in accordance with recommendations, at least 30 ml·kg^− 1^ of fluids were consumed during the day, and an additional personalized supply for before, during, and after training.

The products allowed and included in the diets of the participants:

• Meat—beef, boiled pork, pork (shoulder, pork neck), smoked bacon, chicken breast, turkey breast, salami;

• Fish—cod fillet, tuna, smoked mackerel, salmon fillet, smoked salmon;

• Eggs and dairy products—cheddar cheese, Gouda cheese, feta cheese, cream 30 and 36%, white cheese cheesecake, parmesan, mozzarella, natural yoghurt;

• Fats—ghee butter, rapeseed oil, coconut oil, olive oil, butter, linseed oil;

• Nuts—pistachio nuts, coconut flour, walnuts, almonds, almond milk, almond flour, coconut milk;

• Vegetables—onion, spinach, garlic, tomatoes, parsley, iceberg lettuce, cucumber, chives, red pepper, broccoli, zucchini, dried tomatoes, cauliflower, mushrooms, tomato concentrate, leek, pickled cucumber, black olives, lamb’s lettuce, arugula;

• Fruit—avocado, strawberries, blueberry, raspberries;

• Other—spices (salt, pepper, hot pepper, sweet pepper, coriander, oregano, basil, herbes de Provence, ginger, curry, Cayenne pepper and others), ground flax, balsamic vinegar, stevia, konjac pasta, baking powder.

The products prohibited and excluded from the diet:

• Cereal products (pasta, cereal, bread, rice, cereal flour—wheat, spelled wheat, rye, oats, millet, quinoa, amaranth and others);

• Other fruits, fruit yoghurt;

• Starchy vegetables (potatoes, sweet potatoes, beets);

• Sweets, sugar;

• Alcohol;

• Ready-made seasoning mixes (source of salt, sugar);

• Soda, carbonated, colored drinks, juices, isotonic drinks;

• PRO supplements or gainers and other supplements.

Daily diets were divided into 5 moderate-size meals to prevent digestive problems caused by the consumption of large food portions (Additional file 2: Material S2).

All instructions about foods and meal preparation were provided by dieticians. The participants were encouraged to contact the dieticians with any questions or concerns about the diets, as well as to recommend a suitable substitute in terms of composition in the event that there was no possibility of purchasing/preparing a product included on the menu.

For food intake recording, the participants used food diaries and electronic kitchen scales. The dieticians gave instructions to each participant individually on how to use the scales and complete the diaries. Participants recorded the time and amount of foods and beverages consumed at each meal, the amount of leftovers, and any deviations from the diet.

The diaries were collected at the T_1_ and T_2_ visits. A dietician reviewed and discussed the diary with each participant. The energy and nutrient intakes were calculated with Dietetyk-2 software (JuMar 2016, Poznań, Poland).

The participants were also encouraged to contact the dieticians or the other research team members if any side effects of KD occurred, e.g., nausea, gastrointestinal problems (constipation or diarrhea), irritability, or fatigue. Additionally, they were also asked about potential side effects during KD and at the T_2_ visit.

### Anthropometry and body composition

Body mass and height were measured each time the participants visited the laboratory (T_1_ and T_2_) in duplicate using a calibrated scale with a stadiometer (WPT 60/150 OW, Radwag®, Radom, Poland) in a fasted state to the nearest 0.1 kg and 0.1 cm, respectively. Fat-free mass and fat mass were assessed by air displacement plethysmography (Bod Pod®, Cosmed, Rome, Italy) [51]. The total body water and hydration level were assessed by bioelectric impedance with Bodystat 1500 (Bodystat Inc., Douglas, UK), and via urine specific gravity measurement with URYXXON® Relax (Macherey-Nagel, Düren, Germany); values < 1.020 indicated proper hydration. During the bioimpedance analyses, the recommended measurement conditions were strictly followed [52].

### Daily energy expenditure

The total daily EE was assessed as described previously using heart rate (HR) monitoring data (Polar RS-400, Vantaa, Finland), based on a previously validated method [50, 53, 54]. During run-in phase, each participant’s HR was recorded minute-by-minute for five consecutive days. To eliminate any accidental errors (e.g., cell phone interference, loss of skin contact during sleep) in HR recordings, participants were asked to report the times and types of habitual daily activities and training. The information was used to fill the potential gaps in HR recordings. Recorded wrist-worn HR data were downloaded to a computer equipped with the Polar ProTrainer 5 program (ver. 5.41.002, Vantaa, Finland). On a T_0_ visit, the HR thresholds (HR_FLEX_) for activity categories (sedentary, light, moderate, and vigorous) were estimated individually for each participant. The EE for each category was calculated as recommended, and four-day HR data were categorized into intensity levels and used to estimate the total daily EE [50, 53, 54].

### Blood and urine sample analysis

In order to obtain confirmation of compliance with the KD regime by the subjects, the KB concentration was assessed in urine and blood samples. Fingertip blood and urine samples were taken pre-exercise at T_1_ and T_2_ (~ 3 h post prandial). All blood samples were taken with the patient in a seated upright position. β-hydroxbutyrate (βHB) concentrations were determined with a handheld Optium Xido Neo analyzer (Abbott Diabetes Care Ltd., Witney, Oxon, UK) and Optium Xido β–ketone testing strips (Abbott Diabetes Care Ltd., Witney, Oxon, UK). The apparatus measurement accuracy (CV) was 3.8%. Furthermore, the KB concentration in the urine was examined using the URYXXON® Relax analyzer (Macherey-Nagel, Düren, Germany). The sensitivity settings were adjusted into four ranges (NEG: < 25 mg·dL^− 1^; +: > 25 mg·dL^− 1^; ++: > 100 mg·dL^− 1^; and +++: > 300 mg·dL^− 1^).

A βHB concentration of 0.5–3.0 mmol·L^− 1^ in the blood and KB > 25 mg·dL^− 1^ in the urine used as confirmation of proper adherence to the diet by the subjects [3, 55, 56].

### Exercise tests

Throughout the study protocol, the subjects performed two exercise tests (T_1**–**2_). The incremental cycling tests (ICT) were performed under standardized conditions (temp. 20–22 °C and humidity 60–70%) in the morning (from 8.00 to 12.00 a.m.). Prior to each test, the subjects were instructed about its procedure and completed a brief 5-min warm-up on a cycloergometer (Kettler-X1, Kettler, Ense-Parsit, Germany) (Fig. 1). The aerobic capacity and level of exercise intensity at different ICT stages were evaluated based on the subject’s maximal oxygen uptake (VO_2max_). Following ~ 5-min warm-up on the cycloergometer (Kettler-X1, Kettler, Ense-Parsit, Germany) followed by ~ 5 min rest and preparation for exercise testing, the ICT began at a workload of 75 W for females and 100 W for males at 70 ± 5 rpm (Fig. 1). Every 1.5 min, the workload increased by 25 W until reaching maximum perceived exhaustion, assessed using the Borg scale (6–20) [57, 58]. The respiration indices were recorded with a calibrated ergospirometer (Quark CPET, Cosmed, Rome, Italy) and analyzed with Cosmed CPET software (ver. 9.1b, 2010). VO_2max_ was defined as the point where a workload increase stopped generating further increases in oxygen uptake (VO_2_) and HR [57, 58]. Although all participants were familiar with the tests from T_0_ as well as from previous studies and training, they were encouraged to ask questions about the details of the protocol. Furthermore, the subjects were asked to wear proper workout clothing and shoes. All testing was supervised by an experienced researcher.

### Statistical analysis

The results are presented as means ± SD or ± 95% confidence intervals (CI). Due to significant differences in body composition in females and males, all the results (substrate oxidation rate and EE) are given in relation to fat free mass (FFM) content. The area under the curve (AUC) was calculated using the trapezoid rule for EE and fat and CHO oxidation. The differences between pairs of observations for the rates of substrate utilization and energy expenditure (at separate VO_2max_ points in the range of 30 to 100% of VO_2max_) were tested using repeated measures ANOVA (for normally distributed variables) with post hoc analysis using Scheffe’s test or the Wilcoxon test (for variables without normal distribution of the data after Box-Cox transformation). Either the one-side paired t-Student test or the Wilcoxon test (depending on the distribution of data—normal or not normal) was used to compare the CD-KD (at the CD and after KD) differences in AUCs, the AUC at ≤65% VO_2max_ and the AUC at > 65% VO_2max_ were assessed. The t-Student test for independent variables or the Mann–Whitney U test was implemented to test gender differences for the rates of substrate utilization and EE (at separate VO_2max_ points in the range of 30 to 100% of VO_2max_) between CD and KD, as well as to test differences in percentages of change in AUCs (AUC ≤65% VO_2max_ and AUC > 65% VO_2max_) between females and males. The chi-square test of independence was implemented to verify the relationship between gender and response to KD (increase/decrease in fat/CHO oxidation). Statistical significance was set at *p <* 0.05. Data were analyzed using the STATISTICA 12 (StatSoft Inc., Tulsa, OK, USA) software program.

Based on other studies that have evaluated nutritional interventions with KD on energy metabolism during exercise, a sample size of eight to eleven per group was deemed to be common [41–45, 59]. Therefore, an initial sample size of 11 per group (females and males) was selected. Furthermore, to confirm that this would be sufficient to detect an intervention effect, a statistical power analysis was performed (G*Power, version 3.1.9.2, Universität Düsseldorf, Germany). The power analysis was performed based on the following inputs: t-test differences between two dependent means (matched pairs) or ANOVA repeated measures between-factors; an alpha value of 0.05, a desired power of 0.80; and a sample size of 11 subjects in each group. The results indicated that a standardized effect size of 0.63 or 0.54 would be detectable (large effect). Furthermore, based on our pilot study and previous studies on different dietary regimes on energy metabolism [50], we assumed that a sample size of 11 in each group (females and males) would be sufficient to detect significant differences in fat oxidation.

## Results

### Participants and adherence

There were no differences in energy intake or PRO intake at the CD and during the KD (Table 2). Fat intake was significantly higher and CHO intake was significantly lower during the KD compared to the CD in both females and males. The compliance with the prescribed KD was high and intakes of energy and macronutrients did not differ between prescribed diets and data from dietary records (all *p* > 0.05; data not presented). Also, the analysis of the concentrations of βHB (1.3 ± 0.9 mmol·L^− 1^ in females and 1.2 ± 0.5 mmol·L^− 1^ in males in blood samples, respectively) and KB (in all subjects > 25 mg·dL^− 1^ in urine) confirmed compliance with the diet. Importantly, in the context of results interpretation, we did not observe significant changes in VO_2_max under KD (Table 1).

Furthermore, despite the low supply of fiber in the diet (about 9–10 g·day^− 1^), the subjects did not report gastrointestinal problems, constipation, or diarrhea. On the other hand, reported inconveniences resulting from the conversion of energy processes related to the use of glucose in favor of KB were irritability, drowsiness, and changes in mood. In some of the subjects, none of the above ailments occurred, while in athletes who had difficulty with ketoadaptation, the symptoms lasted from a few days to a maximum of a week. This aspect was also one of the reasons for the resignation of some study participants. Apart from low supply of fiber, the KD during the study protocol was characterized by high nutritional value with regard to vitamins and minerals. The intake of most of them did not changed or was even higher during KD. The only exception was Mg, intake of which was lower during KD compared to CD (Additional file 1: Table S1).

From 30 participants, eight did not complete the study either due to injury (1 female and 1 male) or non-adherence to the protocol and failure to achieve a state of ketosis (2 females and 4 males) (Fig. 1). Twenty-two participants (11 females and 11 males) met the criteria required in the study protocol, completed the nutritional intervention periods and two visits (T_1–2_), and reported no significant lifestyle and training routine changes during the study.

### Fat and carbohydrate oxidation during the customary diet and after the ketogenic diet

**Fig. 2:**
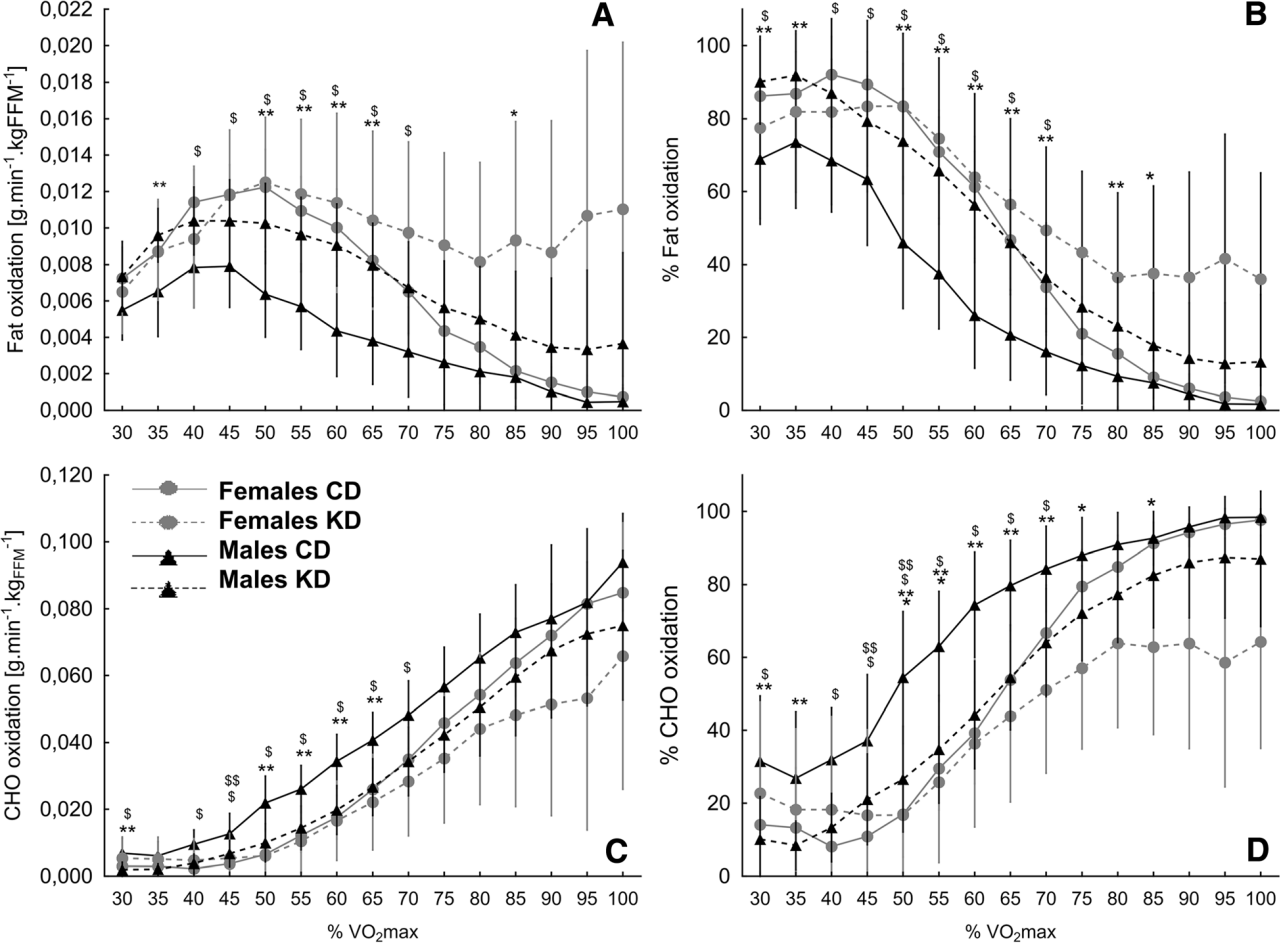
Fat and CHO oxidation rate (**a** and **c**) and their contribution to energy metabolism (**b** and **d**) at the CD and after the KD*. Note: Values are means ± 95% CI*. *Significantly different from CD for VO_2_max points treated separately in females; **Significantly different from CD for VO_2_max points treated separately in males; ^$^significantly different between females and males at CD; ^$$^significantly different between females and males at KD; (*p* < 0.05)

In females, the rate of fat oxidation (g·min^− 1^·kg_FFM_^− 1^) and the percentage contribution of fat oxidation to the energy yield (% oxidation) were noticeably higher during the KD starting from 60% of VO_2max_ (Fig. 2a-b). However the differences between CD and KD reached statistical significance solely at 85% of VO_2max_. In males, the rate of fat oxidation during the KD was higher throughout the whole exercise as compared to CD testing (Fig. 2a), and the differences were significant at 35% and 50–65% of VO_2max_. Similarly, the %fat oxidation was also higher at each separate %VO_2max_ point, but the differences were significant at 30–35, 50–70 and 80% of VO_2max_ (Fig. 2b).

Moreover, in females, starting from about 65% VO_2max_, the rate of CHO oxidation (g·min^− 1^·kg_FFM_^− 1^) at KD assessment was lower compared to during the CD analyses (Fig. 2c), yet the differences did not reach statistical significance at any tested point. While, the contribution of CHO to energy metabolism was significantly lower at KD assessment at 50–55, 75 and 85% of VO_2max_ (Fig. 2d). In contrast, in males, the rate of CHO oxidation at KD testing was lower throughout the whole exercise as compared to during CD testing (Fig. 2c). Still, the differences reached statistical significance only at the lower ranges of VO_2max_ (30% and 50–65% of VO_2max_). Similarly, the contribution of CHO oxidation to energy metabolism during the KD exercise was lower for the whole analyzed range of VO_2max_ (Fig. 2d), and reached significance at 30–35% and 50–70% of VO_2max_.

### Substrate utilization and energy expenditure between females and males

At CD testing, the rate of fat oxidation (g·min^− 1^·kg_FFM_^− 1^) was significantly higher in females at 40–70% of VO_2max_ (Fig. 2a). At KD assessment, despite the apparent differences between females and males starting from 45% of VO_2_max, no significant between-gender differences were noted (Fig. 2a). At CD testing, the percentage of fat oxidation was significantly higher in females at 30 and 40–70% of VO_2max_ (Fig. 2b) but took almost the same values as in men at the highest ranges of VO_2max_ (85–100%). In contrast, after KD, there were no significant between-gender differences at any point of VO_2_max testing (Fig. 2b). However, some between-gender differences occurred at middle and higher levels of VO_2_max (70–100% VO_2_max), the contribution of fat oxidation in energy metabolism was slightly higher in females compared to males.

During CD testing, the level of CHO oxidation (g·min^− 1^·kg_FFM_^− 1^) in males was significantly higher at 30%, and 40–70% of VO_2max_ points, while at the KD assessment, the differences remained significant solely at 45% of VO_2max_ (Fig. 2c). Furthermore, at the CD testing, the contribution of CHO oxidation to the energy yield was significantly higher in males compared to females at 30%, 40–70% of VO_2max_ (Fig. 2d). At the KD testing point, contribution of CHO oxidation was higher at in males starting from 45% of VO_2max,_ still the differences were significant solely at 45–50% of VO_2max_ (Fig. 2d).

**Fig. 3:**
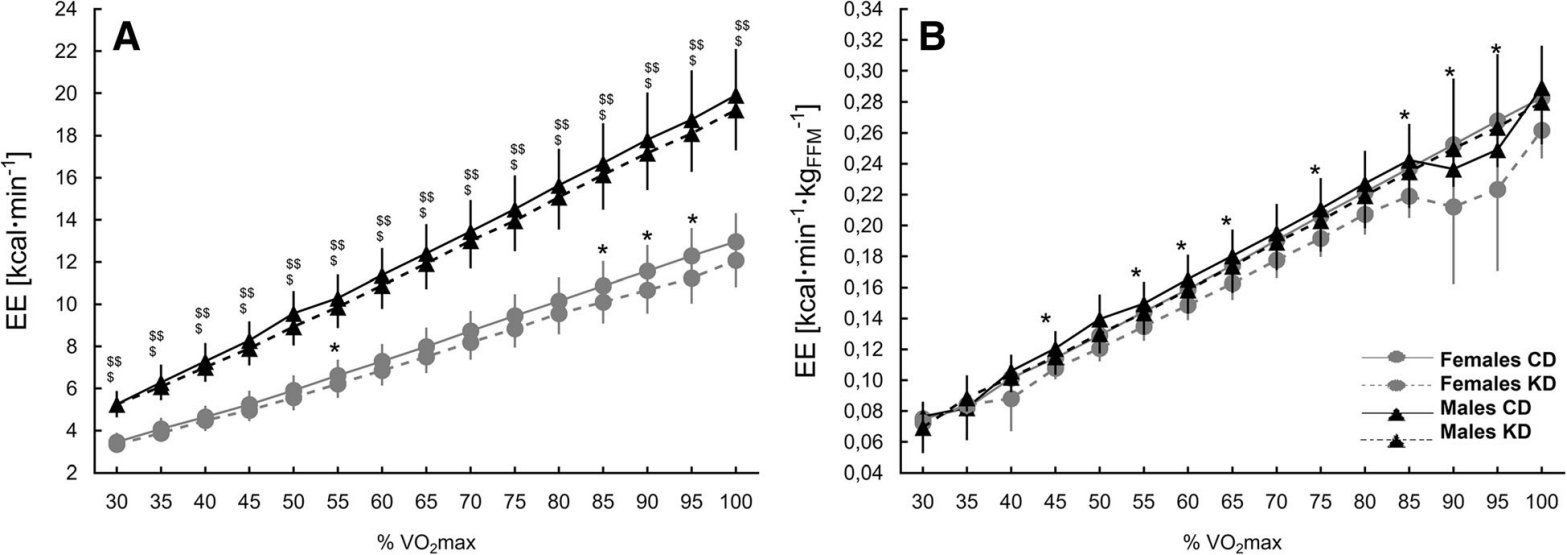
Energy expenditure during exercise at the CD and after the KD intervention. *Note: Values are means ± 95% CI*. *Significantly different from CD for VO_2max_ points treated separately in females (*p* < 0.05)

Although the level of EE (kcal·min^− 1^) seemed to be comparable for the CD and KD analyses, statistically significant differences occurred at 55% and 85–95% of VO_2max_ in females (lower at KD; Fig. 3a). In males EE was significantly higher compared to females at all the analyzed VO_2max_ point during CD and KD testing. While when expressing EE in kcal·min^− 1^·kg_FFM_^− 1^, no differences between females and males were observed at CD or KD testing (Fig. 3b). However, in females EE was significantly lower at 45%, 55–65, 75% and 85–95% VO_2max_ at KD compared to CD.

### Areas under the curve (AUCs) of substrate utilization and energy expenditure during the customary diet and after the ketogenic diet in females and males

**Fig. 4:**
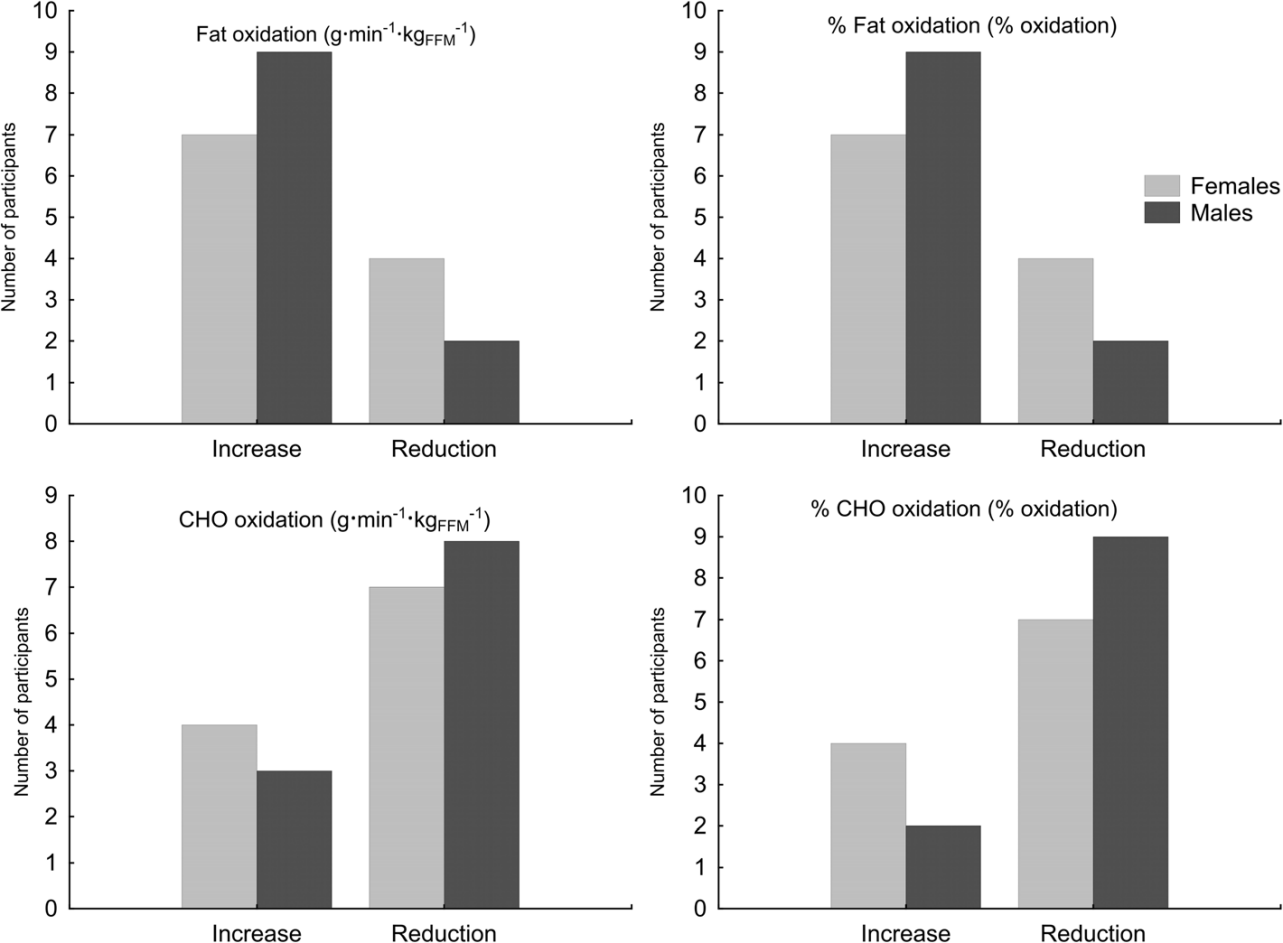
Numbers of female and male participants who experienced an increase or reduction in the AUCs of fat and carbohydrate utilization after the KD diet

In females, there were no differences in the AUCs of fat and CHO oxidation (g·min^− 1^·kg_FFM_^− 1^) or for the percentage contributions of fat and CHO oxidation to energy metabolism between CD and KD (Table 3). However, in females, the AUC of EE (kcal·min^− 1^·kg_FFM_^− 1^) at KD testing was significantly lower compared to CD. In males, the AUCs for the rate of fat oxidation (g·min^− 1^·kg_FFM_^− 1^) and %fat oxidation (% oxidation) were significantly higher at KD compared to CD. There were no differences in the percentage changes of AUCs between females and males for any of measured parameters (Table 3). Furthermore, more males than females showed an increased AUC for fat utilization and a decreased AUC of CHO utilization during KD (Fig. 4). However, the differences were not significant.

**Table 3:** Differences in AUC and percentage changes in AUC at the CD and KD testing points

Indicator	Females CD vs KD *p*	Males CD vs KD *p*	% CD-KD changes females vs males *p*
Fat oxidation (g·min^− 1^·kg_FFM_^− 1^)	*> 0.05*	*< 0.05↑*	*> 0.05*
%Fat oxidation (% oxidation)	*> 0.05*	*< 0.05↑*	*> 0.05*
CHO oxidation (g·min^− 1^·kg_FFM_^− 1^)	*> 0.05*	*> 0.05*	*> 0.05*
%CHO oxidation (% oxidation)	*> 0.05*	*> 0.05*	*> 0.05*
EE (kcal·min^− 1^·kg_FFM_^− 1^)	*< 0.05↓*	*> 0.05*	*> 0.05*

*Note: Data express p-value for differences between CD and KD or females and males*

### AUCs of substrate utilization and energy expenditure according to exercise intensity (≤65% VO_2max_ and > 65% VO_2max_) during the CD and after the KD in females and males

In females, at ≤65% VO_2max_, there were no differences in the AUCs of fat or CHO utilization (oxidation in g·min^− 1^·kg_FFM_^− 1^ and % oxidation) or EE (kcal·min^− 1^·kg_FFM_^− 1^) between CD and KD (Table 4). However, in males, a significant increase and a significant decrease were observed regarding fat and CHO utilization (oxidation in g·min^− 1^·kg_FFM_^− 1^ and % oxidation), respectively. At intensities > 65% VO_2max_, decreases in the AUCs for %CHO oxidation (% oxidation) and EE (kcal·min^− 1^·kg_FFM_^− 1^) were observed in females. No differences were observed in males. No between-gender differences were observed for the percentage changes of AUCs at ≤65% VO_2max_ or > 65% VO_2max_.

**Table 4:** Differences in AUCs and percentage changes in AUCs (exercise intensity: ≤65% VO_2max_ and > 65% VO_2max_) between CD and KD

Indicator	Females CD vs KD		Males CD vs KD		% CD-KD changes females vs males	
	VO_2max_		VO_2max_		VO_2max_	
	≤65%	> 65%	≤65%	> 65%	≤65%	> 65%
	*p*	*p*	*p*	*p*	*p*	*p*
Fat oxidation (g·min^−1^·kg_FFM_^−1^)	*> 0.05*	*> 0.05*	*< 0.05↑*	*> 0.05*	*> 0.05*	*> 0.05*
%Fat oxidation (% oxidation)	*> 0.05*	*> 0.05*	*< 0.05↑*	*> 0.05*	*> 0.05*	*> 0.05*
CHO oxidation (g·min^−1^·kg_FFM_^− 1^)	*> 0.05*	*> 0.05*	*< 0.05↓*	*> 0.05*	*> 0.05*	*> 0.05*
%CHO oxidation (% oxidation)	*> 0.05*	*< 0.05↓*	*< 0.05↓*	*> 0.05*	*> 0.05*	*> 0.05*
EE (kcal·min^−1^·kg_FFM_^− 1^)	*> 0.05*	*< 0.05↓*	*> 0.05*	*> 0.05*	*> 0.05*	*> 0.05*

*Note: Data express p-value for differences between CD and KD or females and males*

## Discussion

In the present study, we investigated the influence of a 4-week KD on fat and CHO utilization and EE during an incremental cycling test in male and female CrossFit practitioners. We demonstrated shifts in macronutrient utilization in favor of fat utilization. Interestingly, there were gender differences in the post-intervention relation between exercise intensity and fat oxidation. For both genders, an increase in fat oxidation during exercise was noticeable. However, in males, an increased rates of fat utilization (oxidation in g·min^− 1^·kg_FFM_^− 1^ and % oxidation) occurred at exercise intensities of up to 80% of VO_2max_ and were statistically significant at 35% and 50–65% of VO_2max_ and 30–35% and 50–70 and 80% of VO_2max_, respectively. In contrast, in females, increased fat utilization (g·min^− 1^·kg_FFM_^− 1^ and % oxidation) was noted at the higher ranges of VO_2max_ (65–100% VO_2max_), but reached statistical significance solely at single points of VO_2max_ (85% of VO_2max_). Gender differences in macronutrient utilization following the KD were particularly apparent when comparing the AUCs of fat and CHO utilization (g·min^− 1^·kg_FFM_^− 1^ and % oxidation) of exercise intensity ≤65% VO_2max_, respectively. In males, the AUC of fat utilization was significantly higher and the AUC of CHO utilization was significantly lower at ≤65% VO_2max_ after the KD compared to the CD. No differences were noted in females.

The authors of this paper are of the opinion that the legitimacy of using the KD is particularly interesting in the increasingly popular disciplines of functional training, such as CrossFit. In this group of athletes, the KD is characterized by a large and constantly growing popularity; however, there is not enough research on its actual impact. Attention is drawn to the fact that in this type of effort, exercises of varying intensities are combined (resistance, speed-strength, and endurance) [60]. The question then arises as to whether the limited availability of CHO and the use of a KD will effectively influence changes in metabolic adaptation in CrossFit training on the basis of variable exercise stimuli. Attention is also drawn to the fact that the nature of the effort may be related to the effective use of the KD. In addition to the known impacts of classical strength training on the activity of the mTORC1/p70S6k/4E-1BP pathway leading, among other factors, to the stimulation of protein synthesis and ribosomal biogenesis as well as increases in muscle mass and strength, high intensity interval training (often used as part of the Crossfit training) also stimulates the activity of AMPK, SIRT 1, and p38 MAPK signaling and the expression of PGC-1α in skeletal muscle [61]. The latter leads, among other effects, to increases in mitochondrial biogenesis and the fatty acid oxidation capacity [61–65]. These aspects seem to explain, in part, the metabolic adaptations induced by this kind of exercise. Furthermore, short-term high-intensity interval training and/or sprint interval training (SIT), as opposed to traditional endurance training, can, in many cases, lead to similar adaptations in human skeletal muscle and exercise performance (but without a suppressive influence on factors such as muscle protein synthesis and strength), which will be even more the case when combining these types of training, as is often observed in CrossFit [61].

Among the factors affecting the rate of fat oxidation during exercise there are training status, gender, acute and chronic dietary intakes, fat mass content, and exercise intensity [66–68]. In general, fat oxidation is the predominant fuel source (%) during exercise at submaximal intensities (< 65% VO_2max_). Increases in exercises intensity that exceed 65% of VO_2max_ induce a shift in energy contribution that favors CHO oxidation [68]. In our study, at the pre-intervention testing stage when participants consumed their CD (with percentage energy from CHO 44.2–45.4%, from fat 36.4–36.9%, and from PRO 17.7–19.4%), we confirmed the abovementioned relations regarding exercise intensity (%VO_2max_) and fat and CHO oxidation rates. These were noted in both females and males. Furthermore, in non-athlete, healthy adults consuming a traditional mixed diet (with energy contributions of 45.4%, 34,3, and 17.3% from CHO, fat, and PRO, respectively), it was previously noted that fat and CHO intakes make modest, but importantly, independent contributions to one’s capacity to oxidize fat during exercise [69].

In recent years, Cox et al. [70] has performed a series of experiments in exercising high-performance athletes after generating acute ketosis through the administration of a ketone ester-based drink. The experiments revealed that ketosis may alter substrate competition for respiration and improve oxidative energy transduction under conditions such as endurance exercise. In this manner, nutritional ketosis may help to unlock greater human metabolic potential which can be suppressed by a high CHO diet [70]. Still, the aforementioned effect needs to be interpreted carefully, while it was achieved after generating acute ketosis by synergistic ingestion of ketone esters and CHO. These conditions cannot be recreated in long-term consumption of KD. It should be also taken into account that in practice the “metabolic” potential may not affect the “performance” potential, which seem to confirm the works in which no effect [71] or even a performance impairment after ketones ester treatment were observed [72, 73]. However, the utility of the KD in athletes has been studied extensively in the last decade [3, 38, 41–46, 59, 74, 75]. Still, there is great diversity in study designs and methodologies, including the use of various sport disciplines (artistic gymnastics [42], taekwondo athletes [43], off-road cyclists [44], endurance trained cyclists and runners [3, 45, 46, 59], race walkers [41], CrossFitters [74], overload training [38]) and lengths of dietary intervention (4 days [75], 3 weeks [41, 43, 45], 4 weeks [42, 44], 8 weeks [38], 10 weeks [46], 12 weeks [74], the use of the KD on a habitual basis [59]), as well as differences in the analyzed outcomes. For this reason, it is difficult to make direct comparisons between our results and results obtained by other authors. Most of the studies included only male subjects [3, 38, 41, 42, 44–46, 59], with only a few studies including both male and female participants [38, 43, 46]. It needs to be emphasized that none of the previous studies focused solely on female athletes or compared the effect of the KD between males and females. For this reason, the between-gender comparison conducted in our study seems to be an original approach and a valuable scientific and practical contribution. Furthermore, CrossFit is still gaining popularity [60], but to our knowledge, there is only one published study on the use of a KD in CrossFit [74]. In the latter study, it was observed that a 12-week KD, in comparison to a control diet, caused marked reductions in whole-body adiposity while not affecting performance indices in recreationally-trained CrossFit trainees. Still, the results of this study need to be interpreted carefully due to its relatively low sample size (7 participants in the KD group and 5 participants in the control group) and lack of randomization of participants into KD or control group (participants were free to choose which diet to follow).

The exercise intensity during CrossFit workouts is estimated to be about 64% of VO_2max_ [76] or 57–66% VO_2max_ [77] depending on training experience, the purpose of training, and for different workouts (‘Cindy’ or ‘Fran’). The increase in fat utilization and in the contribution of fat to energy metabolism expressed by an increase in AUC at intensities ≤65% VO_2max_ in male participants may have led to more optimal employment of energy reserves of the body and hypothetically improved performance during specific efforts in CrossFit workouts. Attempts to evaluate fat utilization and physical performance during discipline-specific efforts have been undertaken by other authors [3, 41, 45, 59]. In those studies KD led to an increase in fat oxidation during exercise, still it was not effective in improving performance in discipline-specific workouts [3, 41, 45, 59]. In a recent, very well controlled study by Burke et al. [41], it was found that KD led decreased performance in elite race walkers by impairing exercise economy and falling into lower end of the intensity spectrum. Thus, there is the need to evaluate the efficacy of the KD, not only with respect to energy metabolism, but also with respect to discipline-specific performance capacity.

Furthermore, the enhancement in fat oxidation under the KD seems to be specific to submaximal intensity exercise. In off-road cyclists, it was noted that the use of a 4-week KD during a period of high-volume low to moderate intensity training led to an increase in the contribution of free fatty acids (FFA) to the total EE during moderate intensity exercise. However, during maximal intensity exercise, FFA metabolism was inhibited, among other factors, by intensified glycolysis [44]. It therefore seems that the KD may also be an inappropriate dietary regime for disciplines characterized by high-intensity, short duration activities relying mainly on the anaerobic energy system. It was previously observed that a 4-day KD resulted in lower peak and mean power during the Wingate anaerobic cycling test, as well as a lower distance run in the Yo-Yo intermittent recovery test compared to high CHO diet [75]. In addition, KD could impair also exercise economy and performance in endurance performance [41].

It should be mentioned here that the duration of the KD may play a crucial role in its efficacy. This is perhaps related to the fact that full ketogenic metabolic adjustment (keto-adaptation) requires about 7 days [42]. Dietary interventions with a KD lasting less than 2 weeks seem to be insufficient to reveal the real effects of ketosis. It is thus crucial to verify that the state of ketosis has been achieved via an evaluation of the level of KB in the urine and/or blood. There are intra-day courses of blood and urinary concentrations of KB during a KD. The highest and most reliably detected levels tend to be observed in the early morning and post-dinner urine [78]. It is thus reasonable to study participants at the exact same time when testing urine or blood ketones.

When analyzing gender differences in substrate oxidation, the way of expressing results – absolute values or in relation fat free mass content, need to be taken into consideration. In a review by Maunder et al. [67], where the fat oxidation was expressed in absolute values (g·min^− 1^) it was concluded that in terms of mixed, mainly high CHO diets, maximal fat oxidation is generally greater in males compared to females, but maximal fat oxidation relative to fat free mass is likely greater in non-obese females compared to non-obese males. It is unknown if this type of metabolic effect would be observed in highly trained individuals. Purdom et al. [68] concluded that women have a significantly greater ability to oxidize fat during exercise. In our study, in general, we observed higher level of fat oxidation in relation to FFM in females compared to males, with significant differences at CD at 40–70% VO_2max_. It seems likely that the gender differences in fat oxidation during exercise can be attributed to the level of circulating estrogen. Estrogen directly stimulates AMPK and PGC-1α, which is thought to increase the downstream fatty acid oxidation protein CD36 and the beta-oxidative protein HAD [68]. Furthermore, beta-oxidative proteins that oxidase long chain fatty acids have been shown to be regulated, in part, by estrogen. The increase in beta-oxidative proteins is directly related to the increased potential of fatty acid oxidation [68]. Circulating estrogen is naturally higher in pre-menopausal women compared to men. There are fluctuations in the level of circulating estrogen throughout the menstrual cycle. The link between estrogen and substrate oxidation, which is reflected in increased fat oxidation rates and decreased CHO oxidation rates during exercise, has been shown by studies in men who received estrogen treatment [79]. However, there are also reports suggesting that elevated estrogen during the follicular phase does not affect fat oxidation when compared to the luteal phase [68]. As it was mentioned before, this is the first study to compare the effects of a KD between females and males. On the basis of our results, it could be stated that male CrossFit practitioners, despite the lower regular rate of fat oxidation during exercise, are more prone to shifts in macronutrient utilization during exercise under the KD than their female counterparts. In males there were more evident changes in fat and CHO utilization after KD, while in females the differences were apparent, but not significant. It is highly interesting that in males, the tendency of increased fat utilization was noticeable at the whole analyzed range of VO_2max_, while in females, the tendency was noted at a higher range of VO_2max_ (65–100% VO_2max_). In a case study by Zinn et al. [46], in which four out of five participants were female, a great increase in the %VO_2max_ at which the peak of absolute fat oxidation occurred was observed after a 10-week KD (48.2% vs 63.7% of VO_2max_). Taking into account our results and the results presented by Zinn et al., it could be hypothesized that, in females, a KD tends to promote a shift of maximal fat oxidation toward higher exercise intensities. It is thus reasonable to consider the effectiveness of KD application not only with regard to the nature and intensity of an effort, but also to consider gender differences in response to the KD at a given exercise intensity in a given sport. It is therefore hypothetically possible that a KD may be beneficial for given types of physical effort in one gender but not the other. Furthermore, as we observed the decreased energetic cost at particular levels of exercise intensity, we believe that the issue demands further insights, with respect to alternations in EE during longer-lasting exercises or training units. The possible alternations may be also conditioned by sex.

However, it should be taken into account that our study has some limitations. Firstly, regarding female participants, the menstrual cycle phase was not monitored by hormonal markers or ultrasonography throughout the study course. Still, none of studied women declared the presence of a menstrual cycle disorder that may have affected levels of circulating estrogen and therefore influenced the rate of fat oxidation. It would be valuable to include monitoring of the menstrual cycle in further investigations. Secondly, the lack of a control group may serve as a slight limitation of our study. However, we believe that in CrossFit practitioners with training experience of at least 2 years, consecutive 4 weeks of training with habitual and constant training load (among others without any specific training micro / macrocycle), will not lead to significant changes in aerobic capacity and alternations in macronutrient utilization during exercise. Another issue is the individual variation in vulnerability to the KD and willingness to continue the KD. We would like to highlight that, in our study, we observed positive responses to the KD (increase in fat oxidation) in nine out of 11 males and in seven out of 11 females. McSwiney et al. [3] noted that a 12-week KD led to improvement in 100 km time trial performance in six out of nine participants (the mean time for the 100 km time trial did not change significantly). In our study, three females and five males dropped out during the diet. In the study by McSwiney et al., five out of 27 participants dropped out because the dietary intervention was too difficult to follow. It is thus suggested that the KD may not be suitable for every athlete and should not be undertaken without consideration of an individual’s dietary preferences [3]. It would be also reasonable in the future investigations to attempt to identify factors determining responsiveness to KD. It could be achieved via evaluation of food preferences, eating behaviors, nutritional value of CD or even genetic diversity in participants who dropped out from the study comparing to those who were able to complete the study protocol. Taking into account aforementioned individual variation in vulnerability to the KD, food preferences of athletes, or type of efforts they are undertaking, it is crucial for athletes to start KD solely under the supervision of experienced dietitians. Eventually, the lack of accounting for ketones while calculating of substrate oxidation [80] may be considered as a slight limitation of the study. However, we intended our results to be referable with results from other authors. An indisputable strength of our study is the double-checking of achievement of a ketosis state in our participants (whose achievement determined final inclusion in the analysis) via βhB blood concentration and urine ketone level. Compliance with the study protocol in regard to diet and training was also evaluated during face-to-face meetings with study dietitians and CrossFit coaches at clubs as well as via e-mail and phone consultations. Finally, in our study, the effect of the KD was assessed only with regard to the incremental cycling test. Although the results are promising, indicating that the consumption of a KD by male CrossFit practitioners may metabolically lead to more optimal fuel utilization during submaximal exercise, its efficacy during specific workout efforts (typical for the discipline) should be evaluated.

Future research should focus on better understanding gender differences and the influence of female sex hormones on the response to the KD in a larger population. Furthermore, future methodologies should include the effect of KD on performance during discipline-specific workouts.

## Conclusions

Compared to a baseline customary diet, a 4-week ketogenic diet resulted in greater fat oxidation and lower carbohydrate oxidation at exercise intensities ≤65% VO_2max_ in males. In females, a tendency towards increased fat oxidation at exercise intensities > 65% VO_2max_ was observed. Still, males seem to be more prone to having shifts in macronutrient utilization during exercise when following the KD. The presented data on metabolic adaptation to KD, seems to indicate, that CrossFit athletes may effectively adapt to training at wide, but gender-dependent range of exercise intensities. However, these observations should be taken with caution, because in practice the metabolic adaptation may not support the exercise performance, especially in high-intensity disciplines like CrossFit.

### Novelty statement

The novelty of our approach are between gender comparisons of the influence of ketogenic diet on possible differences in energy substrates metabolism at different exercise intensities in actively training male and female CrossFit athletes. These findings could serve as guidance for trainers and athletes who consider achieving their dietary goals based on the ketogenic diet.

## Additional files

Additional file 1: **Table S1.** Detailed nutritional value of customary and ketogenic diets (https://figshare.com/s/4f9dd5d1a6c706c49d9e; doi: https://doi.org/10.6084/m9.figshare.7539233). (PDF 112 kb)

Additional file 2: **Material S2.** Sample Menu (https://figshare.com/s/a7603768f47bcc86dd31; doi: https://doi.org/10.6084/m9.figshare.7539236). (XLSX 18 kb)

## Abbreviations

**↑**: significantly higher compared to baseline
**↓**: significantly lower compared to baseline
AUC: area under the curve
BM: body mass
CD: customary diet
CHO: carbohydrate
CI: confidence intervals
EE: energy expenditure
FFA: free fatty acids
FFM: fat free mass
HR: heart rate
ICT: incremental cycling test
KB: ketone bodies
KD: ketogenic diet
NS: statistically not significant
PRO: protein
SD: standard deviation
T_0_: familiarization visit
T_1_: first series of test procedures
T_2_: second series of test procedures
VO_2_: oxygen uptake
VO_2_max: Maximal oxygen uptake
βHB: β-hydroxbutyrate
