# Supplementary material for: Effect of a four-week ketogenic diet on exercise metabolism in CrossFit-trained athletes
Source: J Int Soc Sports Nutr. 2019 Apr 5;16:16. doi: 10.1186/s12970-019-0284-9 (PMC6451242; doi:10.1186/s12970-019-0284-9)
Supplement: Supplementary file 1 — Table S1. Detailed nutritional value of customary and ketogenic diets (https://figshare.com/s/4f9dd5d1a6c706c49d9e; doi: https://doi.org/10.6084/m9.figshare.7539233). (PDF 112 kb) [file 12970_2019_284_MOESM1_ESM.pdf]

Supplementary Table S1. Detailed nutritional value of customary and ketogenic diets.

		Females	Males
Dietary fiber (g·day^{-1})	Customary diet	32.8 ± 11.0	34.7 ± 6.9
	Ketogenic diet	9.9 ± 2.2*	8.9 ± 1.6*
Cholesterol (mg·day^{-1})	Customary diet	491 ± 292	497 ± 318
	Ketogenic diet	670 ± 509	637 ± 282
Sucrose (g·day^{-1})	Customary diet	43.2 ± 10.5	37.3 ± 11.2
	Ketogenic diet	2.3 ± 0.9*	2.0 ± 1.1*
Ca (mg·day^{-1})	Customary diet	810 ± 387	866 ± 406
	Ketogenic diet	1067 ± 232	1294 ± 450*
Fe (mg·day^{-1})	Customary diet	13.4 ± 2.2	13.8 ± 1.8
	Ketogenic diet	14.1 ± 2.8	15.8 ± 4.9
Na (mg·day^{-1})	Customary diet	1646 ± 556	1457 ± 873
	Ketogenic diet	1796 ± 539	2118 ± 826
K (mg·day^{-1})	Customary diet	3297 ± 1300	3678 ± 1311
	Ketogenic diet	2883 ± 536	2959 ± 518
P (mg·day^{-1})	Customary diet	1248 ± 408	1674 ± 425
	Ketogenic diet	1769 ± 539*	1981 ± 383
Mg (mg·day^{-1})	Customary diet	345 ± 93	435 ± 187
	Ketogenic diet	245 ± 49*	249 ± 58*
Zn (mg·day^{-1})	Customary diet	9.4 ± 1.9	10.7 ± 2.1
	Ketogenic diet	14.2 ± 2.9*	15.8 ± 3.5*
Vitamin A (µg·day^{-1})	Customary diet	1631 ± 863	1386 ± 1047
	Ketogenic diet	2268 ± 716	2486 ± 745*
Thiamine (mg·day^{-1})	Customary diet	0.6 ± 0.2	0.9 ± 0.4
	Ketogenic diet	1.6 ± 0.7*	1.5 ± 0.6*
Riboflavin (mg·day^{-1})	Customary diet	1.2 ± 0.4	1.4 ± 0.6
	Ketogenic diet	2.5 ± 0.4*	3.1 ± 0.7*
Vitamin C (mg·day^{-1})	Customary diet	110 ± 27	207 ± 352
	Ketogenic diet	178 ± 75*	229 ± 86
Vitamin D (µg·day^{-1})	Customary diet	9.3 ± 4.2	9.7 ± 6.0
	Ketogenic diet	17.6 ± 9.5*	17.6 ± 12.3
Vitamin E (mg·day^{-1})	Customary diet	16.8 ± 13.2	20.4 ± 12.5
	Ketogenic diet	29.5 ± 13.6*	29.6 ± 12.2
Niacin (mg·day^{-1})	Customary diet	23.5 ± 12.1	27.3 ± 10.5
	Ketogenic diet	23.0 ± 8.7	31.8 ± 9.3
Pyridoxine (mg·day^{-1})	Customary diet	2.6 ± 0.5	2.8 ± 1.2
	Ketogenic diet	2.4 ± 0.9	2.7 ± 0.9
Folate (µg·day^{-1})	Customary diet	252 ± 83	249 ± 106
	Ketogenic diet	637 ± 200*	628 ± 117*
Cyanocobalamin (mg·day^{-1})	Customary diet	0.7 ± 0.2	0.7 ± 0.2
	Ketogenic diet	11.7 ± 6.8*	11.9 ± 3.0*

Values are expressed as means ± SD. * significantly different from customary diet ($p < 0.05$)
